# Supplementary material for: Punicalagin Ameliorates Lupus Nephritis via Inhibition of PAR2
Source: Int J Mol Sci. 2020 Jul 14;21(14):4975. doi: 10.3390/ijms21144975 (PMC7404282; doi:10.3390/ijms21144975)
Supplement: Supplementary file 1 [file ijms-21-04975-s001.pdf]

International Journal of
Molecular Sciences

MDPI

Supplemental Data

Punicalagin ameliorates lupus nephritis via inhibition of PAR2

1. Supplemental Materials

1.1. Cell Proliferation Assays

MTS assay was performed using the CellTiter 96 Aqueous One Solution Cell Proliferation Assay kit (Promega, Madison, WI, USA). Briefly, NIH3T3 cells not expressing functional PAR2 were seeded into 96-well plates at ~ 30% confluence and incubated for 24 h. The cells were treated with punicalin and PCG, and an equal amount of DMSO was added to the control. After 24 h, cell viability was measured using the MTS assay.

1.2. Hematoxylin and Eosin Staining

Heart, liver, and lung tissues of NZB/W F1 mice were fixed in 4% buffered formalin. The formalin-fixed tissue specimens were embedded in paraffin, cut into 4-μm-thick sections, and stained with hematoxylin and eosin (H-E) (Dako, Tokyo, Japan).

1.3. Measurement of Body Weight

Seven-week-old C57BL6 male mice (Nara Biotech, Korea) were adopted for about 1 week. This experiment was approved by the Yonsei University Animal Care and Use Committee (IACUC-A-201706-594-03). All mice were maintained under a 12:12-h light:dark cycle at room temperature and given free access to laboratory chow and water. PCG were dissolved in phosphate-buffered saline (PBS), and mice were intraperitoneally (IP) injected with 10 mg/kg PCG ($n = 3$) once every 2 days for 10 days. Vehicle-treated mice were IP injected with PBS. Body weight changes were measured every 2 days.

1.4. Flow Cytometry

Spleens were isolated from NZB/W F1 mice (at 30 weeks of age), and single cell suspensions were generated. RBCs were removed using the RBC lysis buffer (Biolegend, San Diego, CA, USA). Splenocytes (1×10^6) were stimulated with anti-CD3 (2 μg/mL) and anti-CD28 (2 μg/mL) for 72 h. For intracellular cytokine staining, cells were re-stimulated with phorbol 12-myristate 13-acetate (PMA, 50 ng/mL; Sigma-Aldrich, St. Louis, MO, USA) and ionomycin (750 ng/mL; Sigma-Aldrich) for 4 h in the presence of GolgiPlug (containing Brefeldin A; BD Biosciences, San Jose, CA, USA). For surface staining of $CD4^+$ T cells and $CD19^+CD138^+$ B cells, the cells were washed and stained with FITC-conjugated anti-mouse CD4 (GK1.5; BD Pharmingen; 1:100 dilution), CD19 (1D3; BD Pharmingen; 1:100 dilution), and Brilliant Violet 605™ anti-mouse CD138 (Biolegend) antibodies, and incubated in ice for 30 min. Subsequently, cells were fixed and permeabilized with a Cytofix/Cytoperm solution (BD Bioscience). Intracellular cytokines were detected using the following antibodies: PE-Anti-Mouse IFN-γ (XMG1.2; BD Pharmingen; 1:100 dilution), PerCP-Cy5.5- Anti-Mouse IL-4 (11B11; BD Pharmingen; 1:100 dilution), APC-Anti-Mouse/Rat IL-17A (ebio17B7; eBioscience, San Diego, CA, USA; 1:100 dilution), PE-Anti-Mouse/Rat Foxp3 (NRRF-30; eBioscience; 1:100 dilution). Intracellular Foxp3 staining was carried out according to the manufacturer's protocols using a Foxp3 staining kit (eBioscience). Flow cytometry data for staining were analyzed using FACS Verse (BD Biosciences), and the cell populations were quantified using the FlowJo V10 software (Tree Star, Ashland, OR, USA).

1.5. Study Approval

All experimental procedures were approved by and followed the guidelines of the Institutional Animal Care and Use Committee (IACUC) of Yonsei Laboratory Animal Research Centre (YLARC). The YLARC-IACUC guidelines for the ethical use of animals (YLARC 2017-0043) were also adhered to.

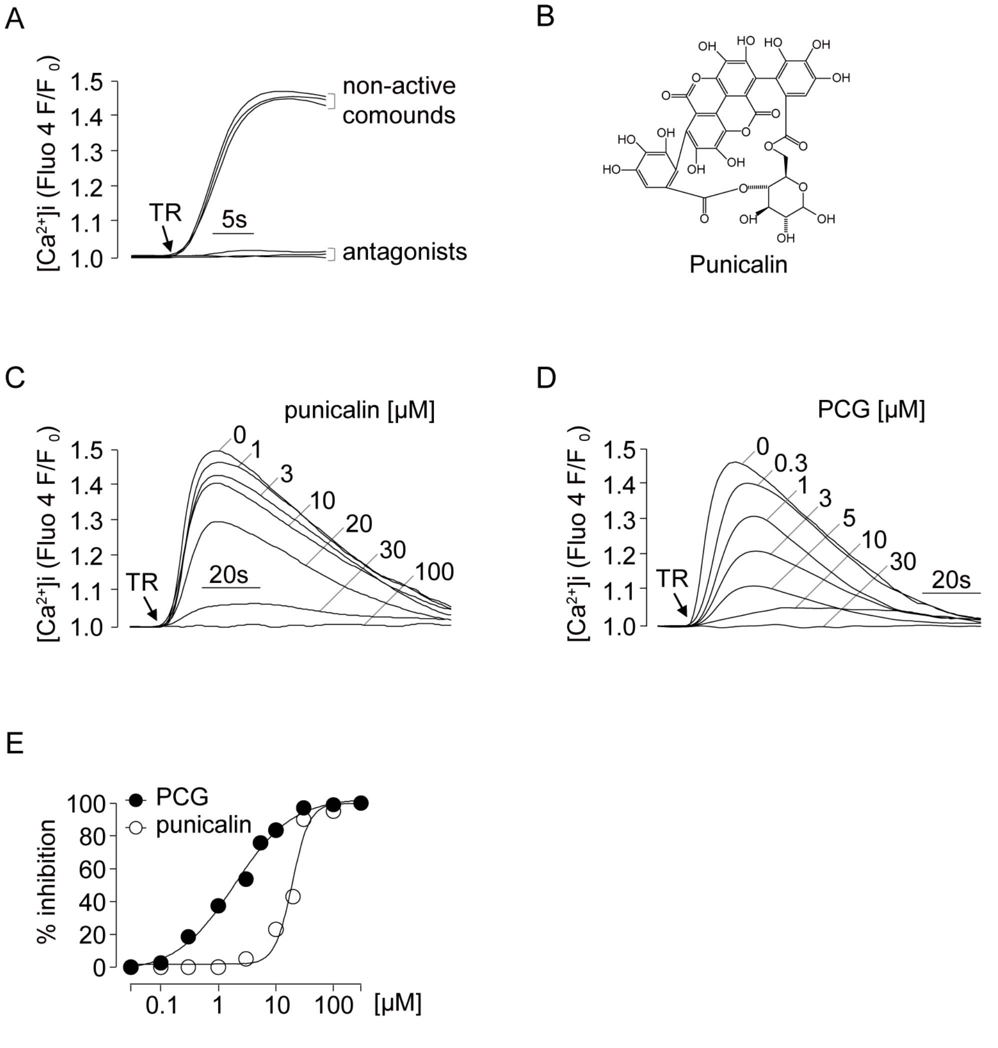

Figure S1. Inhibition of PAR2 by punicalin and PCG in HaCaT cells. (**A**) Representative traces of the intracellular calcium responses of HaCaT cells. PAR2 was activated by 5 nM trypsin (TR). (**B**) Chemical structure of punicalin. (**C**,**D**) Fluo-4 fluorescence intensities were measured in HaCaT cells. Cells were treated with the indicated concentrations of punicalin and PCG 20 min prior to the activation of PAR2 by trypsin. (**E**) Summary of dose responses (mean ± SEM, n = 5–6).

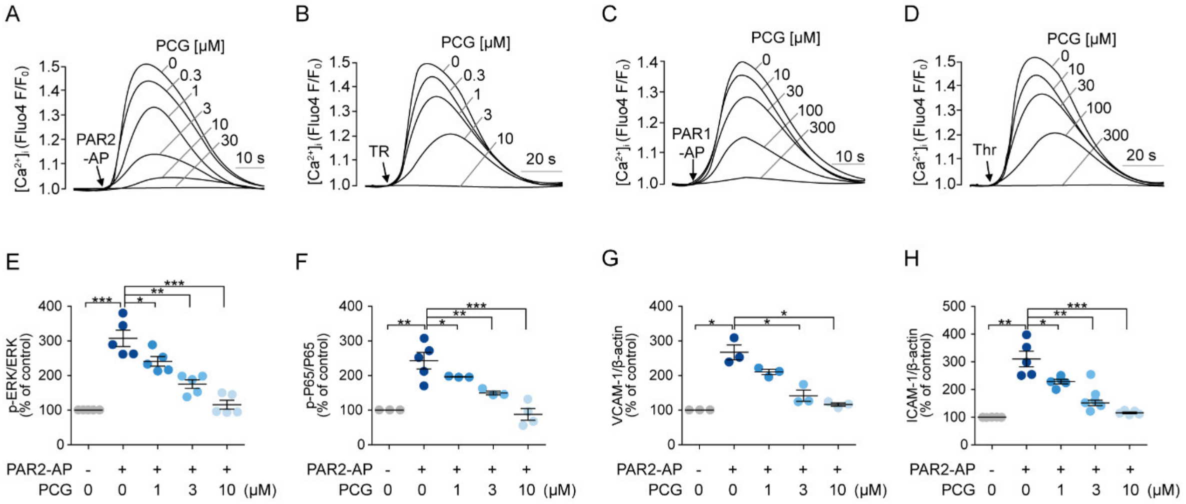

Figure S2. Selective effect of PCG on PAR2, PAR2-induced activation of ERK1/2 and NF-κB, and on the upregulation of VCAM-1 and ICAM-1. (**A**–**D**) Fluo-4 fluorescence intensities were measured in a human podocyte cell line expressing PAR2. The indicated concentrations of PCG were applied 20 min prior to the application of PAR2-AP, trypsin (TR), PAR1-AP, and thrombin (Thr). (**E**,**F**) Inhibition of PAR2 stimulation-induced phosphorylation of ERK1/2 and P65 by PCG. The indicated concentrations of PCG were applied 30 min prior to PAR2 activation by PAR2-AP. The band intensity was normalized against total ERK1/2 and P65 (mean ± SEM, $n = 5–6$). (**G**,**H**) Inhibition of the PAR2-induced upregulation of VCAM-1 and ICAM-1 by PCG. VCAM-1 and ICAM-1 expression levels were detected at 6 h after PAR2 activation. The band intensities of VCAM-1 and ICAM-1 were normalized to that of β-actin (mean ± SEM, $n = 4–6$). *$p < 0.05$, **$p < 0.01$, and ***$p < 0.001$. Two-tailed Student's *t*-test (**E**–**H**).

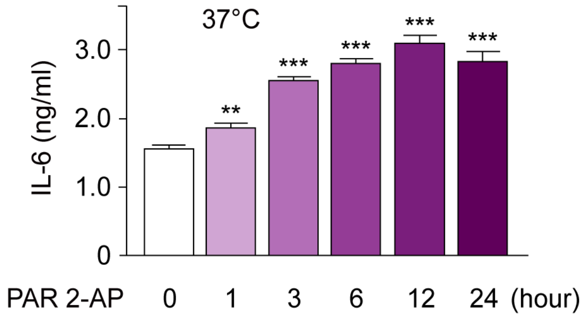

Figure S3. Increase in the production of IL-6 by PAR2 activation in human podocytes. Human podocytes were treated with 10 μM PAR2-AP for the indicated time periods at 37°C; then, IL-6 levels in the medium were determined (mean ± SEM, $n = 3$). **$p < 0.01$ and ***$p < 0.001$. Two-tailed Student's *t*-test.

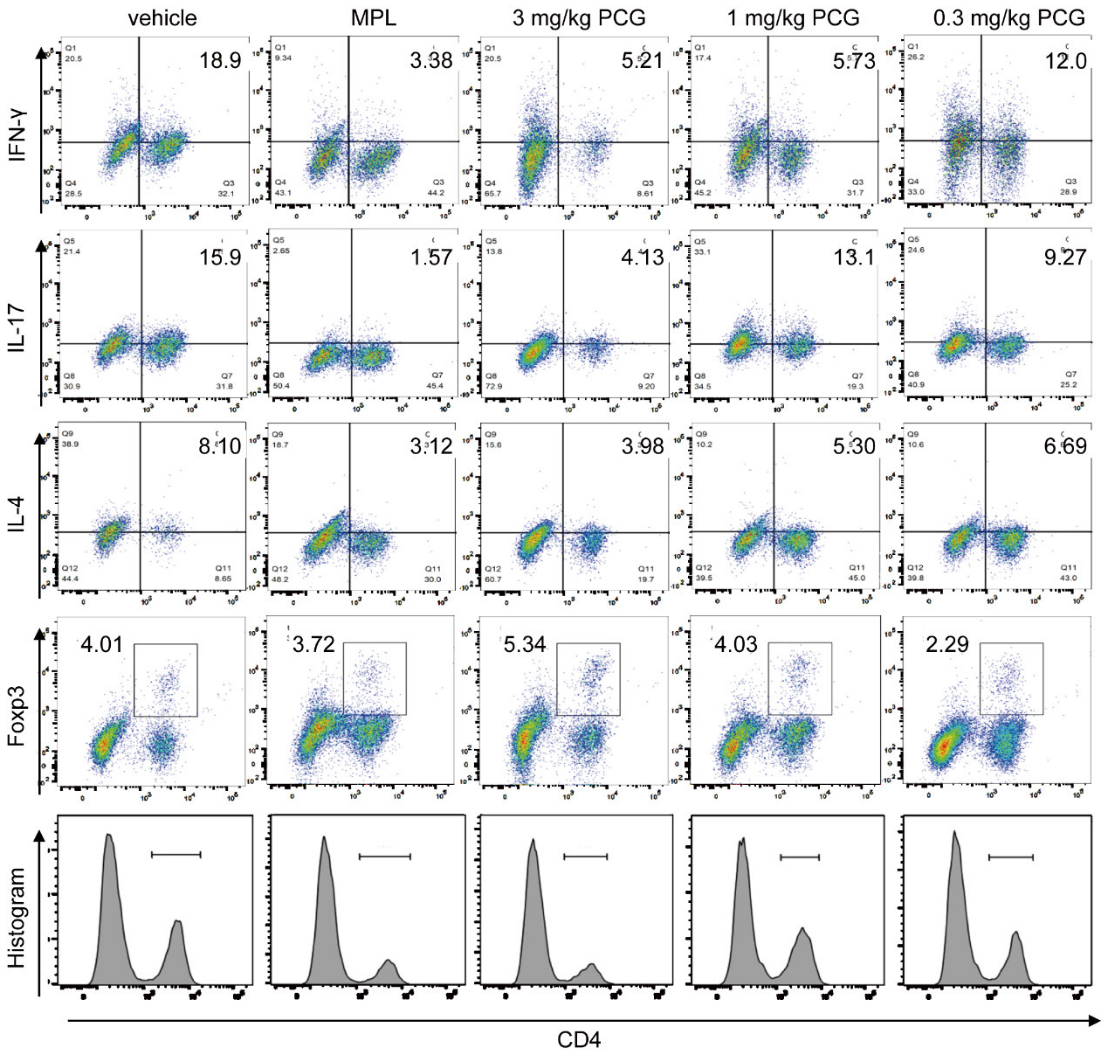

Figure S4. Functional rebalance of T cell subsets by PCG in the spleen. Splenocytes of NZB/W F1 mice were isolated and activated with plate-bound anti-CD3/CD28 for 72 h. Splenocytes were treated with MPL and PCG 3 times a week. The number of $CD4^+$ T cells expressing IFN-γ, IL-17A, IL-4, and Foxp3 were detected by flow cytometry.

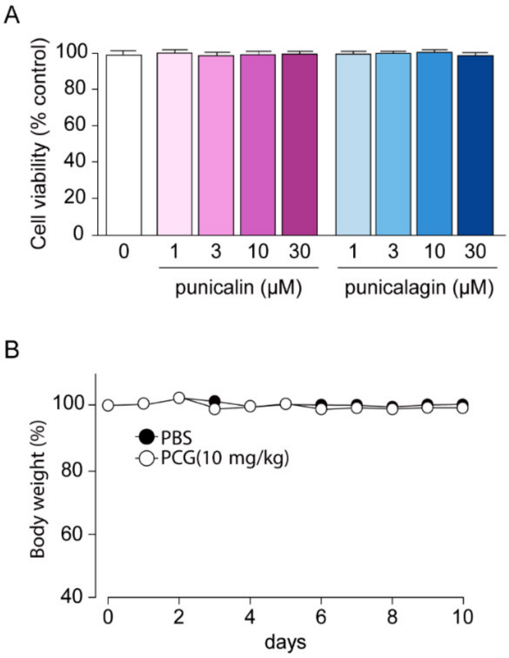

Figure S5. Effect of PCG on cell viability and bodyweight in mice. (**A**) Cell viability was measured in NIH3T3 cells not expressing PAR2. The cells were treated with indicated concentrations of punicalin and PCG and, then, cell viability was determined using the MTS assay after 24 h of incubation (mean ± SEM, n = 3–4). (**B**) Male 8-week-old C57BL6 mice were intraperitoneally injected with PBS (closed circles) or 10 mg/kg of PCG (open circles) every 48 h for 10 days. Body weight changes were observed every 2 days (mean ± SEM, n = 3).

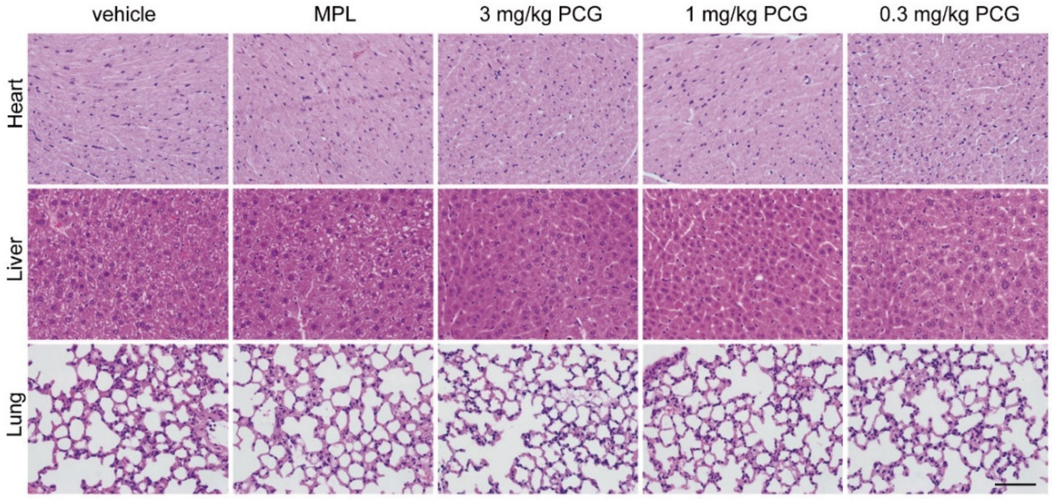

Figure S6. Effect of PCG on heart, liver, and lung tissues of NZB/W F1 mice. Histological sections of major organs, including heart, liver, and lung, were obtained at 30 weeks after intraperitoneal injection of vehicle, MPL, and PCG. Hematoxylin and eosin staining was conducted and images were acquired through light microscopy. Scale bar = 50 μm.

© 2020 by the author. Licensee MDPI, Basel, Switzerland. This article is an open access article distributed under the terms and conditions of the Creative Commons Attribution (CC BY) license (http://creativecommons.org/licenses/by/4.0/).
